# Impact of dietary counseling on Mediterranean diet principles on dietary fiber intake and serum uremic toxins in patients treated with peritoneal dialysis: A pilot study 

**DOI:** 10.5414/CNP104S05

**Published:** 2025-11-28

**Authors:** Kaja Pajk, Nina Bremec, Aljoša Kuzmanovski, Jelka Lindič, Jernej Pajek, Bojan Knap

**Affiliations:** 1 Department for food science, Biotechnical Faculty, University of Ljubljana,; 2 Dietetics and Nutrition Service,; 3 Department of Nephrology, University Medical Center Ljubljana, and; 4 Faculty of Medicine, University of Ljubljana, Ljubljana, Slovenia

**Keywords:** Mediterranean diet, peritoneal dialysis, dietary fiber, uremic toxins

## Abstract

Introduction: The Mediterranean diet (MD) offers numerous health benefits, including improvements in cardiovascular health, cognitive function, and reduced inflammation. Its role in patients with chronic kidney disease (CKD), particularly those undergoing peritoneal dialysis (PD), remains understudied, but attractive. This study evaluated achieved adherence to the MD, impact on dietary fiber intake, and serum uremic toxins in PD patients. Materials and methods: An interventional randomized pilot study was conducted on 21 PD patients, randomized into intervention (MD diet counseling) and control groups (standard diet). Dietary intake, fiber consumption, serum potassium, phosphate and serum uremic toxins (trimethylamine-N-oxide (TMAO), p-cresyl sulfate (pCS), and indoxyl sulfate (IS)) were measured before and after a 4-week intervention. Dietary adherence was assessed using the Mediterranean Diet Adherence Screener (MEDAS). Statistical analyses compared the changes between groups. Results: Adherence to the principles of MD significantly improved in the intervention group (MEDAS: 6.6 ± 1.0 to 8.8 ± 1.2, p < 0.001). Dietary fiber intake increased modestly but not significantly (16.7 ± 6.7 g/day to 19.8 ± 7.5 g/day, p = 0.374). Serum levels of uremic toxins showed no significant change, while potassium and phosphate levels remained stable. Conclusion: The MD counseling improved dietary adherence to the goals of MD without negatively affecting serum electrolyte and phosphate control in PD patients. No significant changes were observed in serum uremic toxin levels or dietary fiber intake.

## Introduction 

The Mediterranean diet (MD) is based on traditional dietary patterns of populations living in Mediterranean countries and is characterized by a high intake of fruits, vegetables, whole grains, legumes, nuts, and olive oil, alongside moderate consumption of fish, poultry, and dairy products, while limiting red meat and sweets [1]. Numerous studies have demonstrated its beneficial effects on cardiovascular risk, weight management, reduction of visceral fat, and improvement of cognitive function, as well as its protective role against non-communicable chronic diseases, including heart disease, stroke, type 2 diabetes, certain cancers, and Alzheimer’s disease [2]. Plant-based food can be very useful for patients with kidney disease due to renoprotective and cardioprotective effects [3]. Given its positive impact on metabolic health and inflammation, the MD has been increasingly explored as a potential nutritional strategy for patients with chronic kidney disease (CKD), including those undergoing peritoneal dialysis (PD) [4, 5]. 

Patients on PD face unique nutritional challenges, as dialysis itself can alter appetite, fluid balance, and nutrient absorption [6]. Studies suggest that these patients often have insufficient energy and protein intake, consume too few fruits and vegetables, and as a result, fail to meet recommended dietary fiber intake [7]. This dietary imbalance can lead to nutritional deficiencies, systemic inflammation, and an increased risk of cardiovascular complications, which are already elevated in CKD patients [8]. Moreover, the accumulation of uremic toxins such as indoxyl sulfate (IS), p-cresyl sulfate (pCS), and trimethylamine-N-oxide (TMAO) – many of which originate from the gut microbiota and are influenced by dietary composition – has been implicated in the progression of CKD and its associated complications [6]. 

The MD, being rich in fiber, polyphenols, and healthy fats, has been hypothesized to modulate gut microbiota, reduce systemic inflammation, and improve metabolic parameters in patients with CKD [1]. In addition, it provides plant-based sources of protein, which may contribute to lower levels of protein-bound uremic toxins, while maintaining adequate energy and nutrient intake. However, despite its benefits, concerns remain regarding its suitability for PD patients, particularly regarding potassium and phosphorus intake, which must be carefully managed in individuals with impaired kidney function [9]. We are not aware of any interventional studies on providing counseling on the MD and comparing it with no counseling, in patients on PD. 

The aim of this study was to investigate the impact of adherence to a MD on dietary intake, fiber intake, potassium, phosphorus, and uremic toxin levels, as well as markers of systemic inflammation, such as interleukin-6 (IL-6). By evaluating these parameters, we sought to determine whether the Mediterranean diet could be a feasible and beneficial dietary approach for PD patients. 

## Materials and methods 

An interventional randomized study was conducted at the Peritoneal Dialysis Center of the Department of Nephrology, University Medical Center Ljubljana. The study included 26 patients (20 men and 6 women) who were undergoing PD. The exclusion criteria were: Charlson Comorbidity Index (CCI) score ≥ 5 points, fulfillment of the diagnostic criteria of the Global Leadership Initiative for Malnutrition (GLIM) for malnutrition, score of ≥ 10 points on the 14-Item Mediterranean Diet Adherence Screener (MEDAS), peritonitis in the past 6 months, or change in dietary habits in the past 6 months. The randomization of patients was conducted using the online tool Research Randomizer [10]. The exclusion criteria after the intervention were: kidney transplantation, newly developed peritonitis or exit site infection, and transition to hemodialysis treatment. The research was approved by the ethics committee number 0120-258/2022/6. 

The final analysis of the study results included 21 patients (17 men and 4 women). The study lasted for four 4 and included 11 patients in the intervention group and 10 patients in the control group. The intervention group received personalized dietary guidance on adhering to the MD, including recipe examples adapted for PD patients. Dietary intake was assessed using a 3-day food diary in the intervention group before and after the intervention, while the control group followed the previous day’s menu recall method at both time points. Blood samples were collected at baseline and after 4 weeks to measure serum electrolytes, including potassium, phosphorus, sodium, calcium, IL-6 and as well as uremic toxins TMAO, IS, and pCS. Adherence to the MD was assessed using the MEDAS questionnaire [11] before and after the intervention. Statistical analyses were performed to examine changes in dietary intake, body composition, biochemical parameters, and inflammatory markers. Paired t-tests, Mann-Whitney U tests, and regression models were used to evaluate the effectiveness of the dietary intervention. 

## Results 

**Figure 1:**
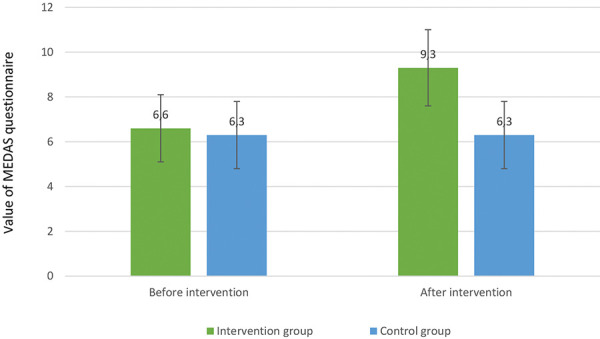
MEDAS adherence scores before and after intervention, showing significant improvement in the intervention group.

The final sample consisted of 17 males (81%) and 4 females (19%), with an average age of 54.9 years (range: 23 – 85 years). The baseline nutritional assessment indicated that 95% of participants consumed lower-than-recommended energy intake (average 1,684 ± 385 kcal) and 33% consumed insufficient protein quantity (on average 0.8 ± 0.3 g/kg BW). 62% of patients were classified as overweight or obese (BMI > 25 kg/m^2^), while 33.3% showed signs of malnutrition, indicated by a low phase angle < 5 degrees on bioelectrical impedance analysis (BIA). The average protein intake before the intervention was below the recommended 1.0 – 1.2 g/kg/day, and fiber intake was generally low. Energy and protein intake post intervention in the interventional group did not show significant changes and remained below the recommended thresholds for both protein (60 ± 25 g before and 62 ± 17 g after) and energy intake (1,748 ± 419 kcal before and 1,747 ± 366 kcal after). Patients in the interventional group significantly improved their adherence to the MD guidelines, as demonstrated by a significant increase in the average MEDAS questionnaire score from 6.6 ± 1.5 to 9.3 ± 1.7 (p = 0.007) (Figure 1). In comparison, adherence scores remained unchanged in the control group. 

**Figure 2:**
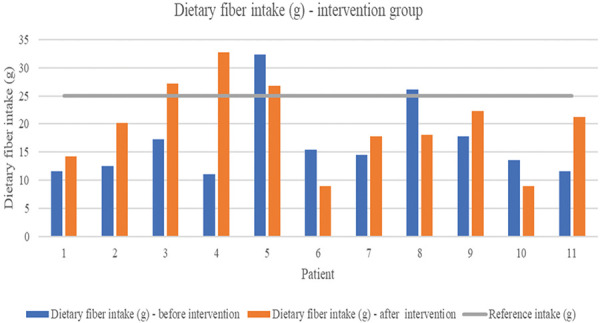
Dietary fiber intake per patient in the intervention group, demonstrating individual variability pre and post intervention.

Dietary fiber intake in the intervention group showed a slight increase from 16.69 ± 6.74 to 19.84 ± 7.45 g/day after 4 weeks of intervention, although this change was not statistically significant (p = 0.374). Figure 2 illustrates the individual changes in dietary fiber intake per patient, highlighting considerable variability among patients, with some exceeding and others remaining below recommended dietary fiber intake levels. 

The analysis of dietary intake included an evaluation of the protein/fiber index (P/F index), reflecting the balance between protein and fiber consumption. At baseline, the P/F index indicated a higher proportion of protein relative to fiber, consistent with known dietary patterns in PD patients. Following the MD intervention, fiber intake increased slightly, while protein intake remained stable, leading to a minor shift in the P/F index. However, this change was not statistically significant. No significant correlations were observed between the P/F index and uremic toxin concentrations (TMAO, pCS, IS). 

**Figure 3:**
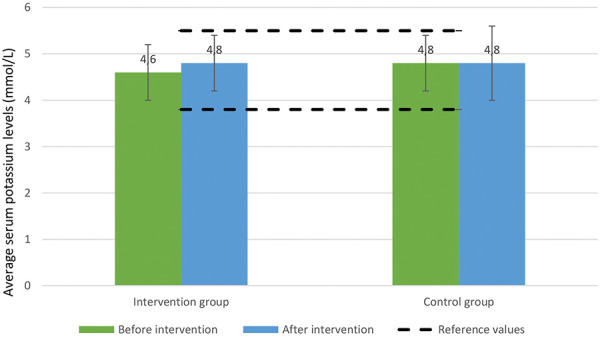
Serum potassium levels before and after intervention.

**Figure 4:**
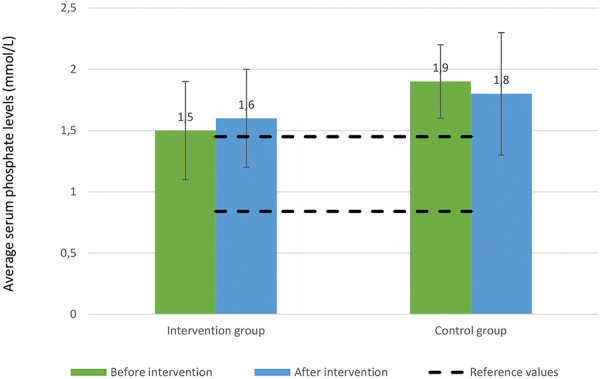
Serum phosphate levels pre and post intervention.

Serum potassium levels remained stable and within reference limits throughout the study period in both groups (Figure 3). Similarly, serum phosphate levels showed no significant alterations, with minor variations slightly above normal reference ranges (Figure 4). 

**Figure 5:**
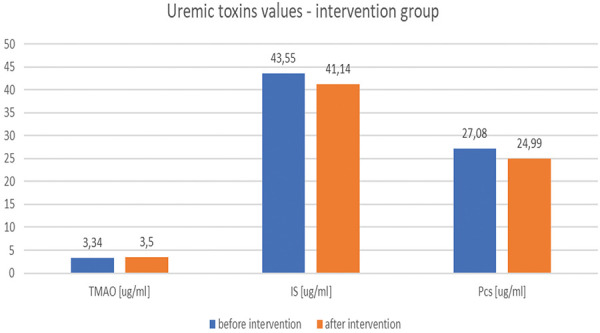
Serum uremic toxin levels (trimethylamine-N-oxide (TMAO), p-cresyl sulfate (pCS), and indoxyl sulfate (IS)) before and after intervention.

Analysis of serum uremic toxins indicated no statistically significant changes within the intervention group (TMAO: from 3.34 ± 1.80 to 3.50 ± 2.64 µg/mL; pCS: from 27.08 ± 18.77 to 24.99 ± 13.97 µg/mL; IS: from 29.63 ± 17.61 to 31.93 ± 27.85 µg/mL) (Figure 5). After 4 weeks of intervention, no statistically significant differences were found in any group in uremic toxin concentrations (TMAO p = 0.552; pCS p = 0.079; IS p = 0.067) (Table 1). 

## Discussion 

This interventional study aimed to investigate the impact of dietary counseling on the principles of MD in patients on PD and comparing this group to no dietary counseling on dietary fiber intake and serum levels of uremic toxins in patients treated with PD. Results from our study indicate significant improvement in adherence to the MD, as confirmed by a statistically significant increase in the MEDAS questionnaire scores (p = 0.007). This suggests that patients undergoing PD can successfully adapt to MD guidelines, given suitable educational support and dietary counseling tailored specifically for this patient population. 

Despite improved adherence, dietary fiber intake increased only modestly in the intervention group (from 16.69 ± 6.74 to 19.84 ± 7.45 g/day) and was not statistically significant. These findings align with previous literature showing PD patients often struggle to reach recommended daily intakes for dietary fiber, primarily due to dietary restrictions and reduced appetite associated with end-stage renal disease (ESRD) and dialysis treatment [12]. Low fiber intake remains a concern due to its implications for gastrointestinal health, potential inflammation, and microbiota dysbiosis, which are particularly problematic in CKD patients undergoing dialysis [13]. 

In our study, despite modest improvements in diet adherence and fiber intake, there were no statistically significant changes in serum uremic toxins – namely TMAO, pCS, and IS. These findings are consistent with other studies that found limited short-term impacts of dietary interventions on these toxins due to complex interactions involving gut microbiota, residual renal function, and dietary protein metabolism [14]. Additionally, no statistically significant differences in uremic toxin levels were observed when comparing intervention and control groups post intervention. It should be emphasized, however, that the duration of the study was short and our sample size was relatively small, which likely reduced the statistical power necessary to detect modest biochemical changes. Regarding serum electrolyte management, the intervention did not adversely affect potassium and phosphate levels, demonstrating that counseling on MD might be safe for PD patients. This is particularly important because diets rich in fruits and vegetables might raise concerns about potassium levels. However, our findings indicate that with proper dietary guidance, the MD can be safely implemented without significant alterations in electrolyte balance. 

The P/F index has been identified as a potential factor in regulating gut-derived uremic toxins in CKD. A lower P/F index, reflecting higher fiber intake relative to protein, has been associated with reduced levels of pCS and IS. In our study, while the MD led to a slight increase in fiber intake, the overall P/F index remained largely unchanged, and no significant reductions in uremic toxin levels were observed [15]. 

The nutritional analysis highlighted that patients generally had inadequate daily energy and protein intake at baseline, which slightly improved after dietary intervention but remained below recommended levels, as previously reported [16, 17]. This finding underscores the persistent nutritional challenges faced by patients undergoing PD, reinforcing the necessity of personalized nutritional counseling as a critical component of their clinical management [18]. Inadequate intake of energy and protein in PD patients contributes significantly to the risk of malnutrition, sarcopenia, and poorer clinical outcomes [19]. Hence, while MD guidelines may enhance diet quality, future studies should consider supplementing the MD with personalized nutritional strategies within MD to ensure adequate energy and protein intake [20]. 

The limitations of this study should be acknowledged, primarily the short duration (4 weeks), small sample size, and inherent variability among participants. A longer study duration or a larger cohort might yield more conclusive evidence on the beneficial effects of dietary interventions. Additionally, future research should also evaluate patient compliance more rigorously, considering factors such as dietary adherence in daily life outside clinical settings, dialysis adequacy, medication use, and the overall physical activity of patients, as these factors may influence outcomes significantly. 

## Conclusion 

Our short-term study showed that MD counseling improved adherence in PD patients without adversely affecting serum electrolyte balance or phosphate concentrations. No significant changes were observed in serum uremic toxin levels or dietary fiber intake. These findings highlight the potential benefits of the MD, underscoring the need for larger, long-term randomized controlled trials to confirm its impact on clinical outcomes. 

## Authors’ contributions 

B.K. conceived and designed the study. N.B., K.P., A.K. helped with the inclusion of patients. K.P. and N.B. performed all the measurements in the study. K.P., N.B., J.L. collected all the data and performed the statistical analysis. K.P., B.K., N.B. wrote the manuscript. N.B., K.P., A.K., J.L., J.P., and B.K. read and critically evaluated the manuscript and gave final approval for publication. 

## Funding 

This research received no funding. 

## Conflict of interest 

The authors declare no conflict of interest. 

**Table 1.** Comparison of uremic toxin and fiber intake levels between intervention and control groups post intervention.

Variable	Group	Mean ± SD^a^	Mann-Whitney U value	p^b^
**TMAO** (µg/mL)	Control group	3.42 ± 1.98	41.000	0.552
	Intervention group	3.50 ± 3.85		
**pCS** (µg/mL)	Control group	36.19 ± 9.98	23.000	0.079
	Intervention group	24.99 ± 13.97		
**IS** (µg/mL)	Control group	48.44 ± 27.14	25.000	0.067
	Intervention group	31.93 ± 27.85		
Dietary fiber (g/day)	Control group	21.14 ± 10.90	48.000	0.941
	Intervention group	19.84 ± 7.45		

^a^Standard deviation; ^b^statistical significance. TMAO = trimethylamine-N-oxide; pCS = p-cresyl sulfate; IS = and indoxyl sulfate.
